# Phytochemical Characterization and Evaluation of the Antioxidant and Anti-Enzymatic Activity of Five Common Spices: Focus on Their Essential Oils and Spent Material Extractives

**DOI:** 10.3390/plants10122692

**Published:** 2021-12-07

**Authors:** Adriana Trifan, Gokhan Zengin, Mihai Brebu, Krystyna Skalicka-Woźniak, Simon Vlad Luca

**Affiliations:** 1 Department of Pharmacognosy, Faculty of Pharmacy, “Grigore T. Popa” University of Medicine and Pharmacy Iasi, 700115 Iasi, Romania; adriana.trifan@umfiasi.ro; 2 Physiology and Biochemistry Research Laboratory, Department of Biology, Science Faculty, Selcuk University, 42130 Konya, Turkey; gokhanzengin@selcuk.edu.tr; 3 Physical Chemistry of Polymers Laboratory, “Petru Poni” Institute of Macromolecular Chemistry, 700481 Iasi, Romania; bmihai@icmpp.ro; 4 Department of Natural Products Chemistry, Medical University of Lublin, 20-093 Lublin, Poland; 5 Biothermodynamics, TUM School of Life and Food Sciences, Technical University of Munich, 85354 Freising, Germany

**Keywords:** antioxidant, anti-enzymatic, spices, aromatic plants, by-products, LC-HRMS/MS

## Abstract

The essential oil industry of aromatic herbs and spices is currently producing a significant amount of by-products, such as the spent plant materials remaining after steam or hydrodistillation, that are simply discarded. The aim of this study was to comparatively investigate the phytochemical composition, antioxidant and multi-enzymatic inhibitory potential of the essential oils and spent plant material extractives obtained from cinnamon, cumin, clove, laurel, and black pepper. The essential oils were characterized by the presence of several phytochemical markers (cinnamaldehyde, cuminaldehyde, eugenol, eucalyptol, *α*-terpinene, limonene, *β*-caryophyllene or *β*-pinene). On the other hand, the LC-HRMS/MS profiling of the spent material extracts allowed the annotation of species specific and non-specific metabolites, such as organic acids, phenolic acids, flavonoids, proanthocyanidins, hydrolysable tannins, fatty acids, or piperamides. All samples exhibited very strong antioxidant effects, with the clove essential oil displaying the strongest radical scavenging (525.78 and 936.44 mg TE/g in DPPH and ABTS assays), reducing (2848.28 and 1927.98 mg TE/g in CUPRAC and FRAP), and total antioxidant capacity (68.19 mmol TE/g). With respect to the anti-acetylcholinesterase (0.73–2.95 mg GALAE/g), anti-butyrylcholinesterase (0–3.41 mg GALAE/g), anti-tyrosinase (0–76.86 mg KAE/g), anti-amylase and anti-glucosidase (both 0–1.00 mmol ACAE/g) assays, the spice samples showed a modest activity. Overall, our study reports that, not only the volatile fractions of common spices, but also their spent plant materials remaining after hydrodistillation can be regarded as rich sources of bioactive molecules with antioxidant and multi-enzymatic inhibitory effects.

## 1. Introduction

Aromatic herbs and spices have been important components of human nutrition since antiquity and are considered as rich dietary sources of phytochemicals for both flavoring and medicinal applications. Aromatic herbs and spices are commonly used in households as culinary ingredients, but their derived essential oils and extracts found various applications within food, confectionery, perfumery, cosmetics, and pharmaceutical sectors [1]. Besides their distinctive flavor profile, aromatic herbs and spices are highly appreciated as natural preservatives in foods due to antioxidant and antimicrobial propensities [2]. Since time immemorial, aromatic herbs and spices have also been employed as traditional remedies in various Asian traditional medicine systems (Chinese, Indian, Korean, and Japanese), which later prompted extensive research concerning their phytoconstituents and potential biological effects [2,3]. Indeed, distinct aroma profiles are attributable to marker compounds such as cinnamaldehyde in cinnamon, eugenol in clove, piperine in black pepper; in addition, various studies showed that these phytochemicals not only give the specific flavor, but also possess significant antimicrobial, antioxidant, anti-inflammatory, antidiabetic, hypolipidemic, and anticancer properties [2,3,4,5]. 

Among spices, cinnamon, cumin, clove, laurel, and black pepper and their derived essential oils are utilized worldwide as flavor, aroma, color, and preservative agents in the food and pharmaceutical industries [3,4]. 

The inner bark of *Cinnamomum verum* J.Presl. (syn. *Cinnamomum zeylanicum* Blume, Lauraceae, cinnamon) is widely used as a spice and flavoring agent in food and industrial products, e.g., baked goods, seasonings, confectionery, candies, chewing gums, drinks, mouthwash, and toothpaste [5,6]. The main compounds identified in cinnamon comprise essential oils (up to 4%), condensed tannins, coumarins, flavonoids, lignans, resins, and sterols [7]. Cinnamon has been used since time immemorial in the treatment of different human ailments, such as respiratory disorders (bronchitis, asthma, fever, common cold, and influenza), gastrointestinal upsets (anorexia, dyspepsia, nausea, flatulent colic, infantile diarrhea), inflammation, and headache [8,9]. Modern studies revealed that cinnamon possesses pleiotropic pharmacological effects, including antimicrobial, anthelmintic, antidiabetic, antitumor, anti-inflammatory, analgesic, antidepressant, and neuroprotective activities [10].

The dried flower buds of *Syzygium aromaticum* (L.) Merr. & L.M.Perry (syn. *Eugenia caryophyllata* Thunb., Myrtaceae, clove) are broadly used as a culinary spice and flavoring agent in foods, perfumery, cosmetics, and in the pharmaceutical industry [11,12]. Various classes of phytochemicals have been reported in clove and refer mainly to essential oils (up to 20%), flavonoids, phenolic acids, ellagitannins, saponins, sterols, and lipids [13,14]. Traditionally, clove is used as a remedy for toothache, mouth and throat inflammation, bronchitis, cough, digestive disorders, rheumatism, and myalgia [15]. The bioactivity of clove extractives has been well-documented and refers to antispasmodic, antifungal, antibacterial, antiviral, antioxidant, anti-inflammatory, antinociceptive, antihistaminic, and anthelmintic properties [3,14,16].

The dried fruits of *Cuminum cyminum* L. (Apiaceae, cumin) are the main components of curry and chili powders, being largely used in bakery, meat products, soups, snacks, and as a preservative in food processing [17]. The traditional uses of cumin relate to alleviation of digestive complaints (dyspepsia, flatulence, colic, and diarrhea) and stimulation of lactation [18]. The main phytoconstituents found in cumin are essential oils (up to 5%), flavonoids, tannins, phenolic acids, and fatty oil [10]. In addition, literature reports show that cumin is endowed with significant pharmacological effects, such as antifungal, antibacterial, antioxidant, antispasmodic, antitumor, anti-inflammatory, hypoglycemic, and hypolipidemic activities [19,20].

The dried leaves of *Laurus nobilis* L. (Lauraceae, laurel) are a popular spice commonly used in the culinary, food, and fragrance industries [21,22,23]. The phytochemistry of laurel is characterized by the presence of essential oils (up to 3%), sesquiterpene lactones, alkaloids, flavonoids, phenolic acids, proanthocyanidins, and fixed oils [24,25]. Since antiquity, laurel has been used against digestive disorders (bloating and flatulence), respiratory infections, cough, and rheumatism [26]. Moreover, recent studies prompted various properties of laurel extractives, e.g., antimicrobial, antioxidant, anti-inflammatory, antidiabetic, insecticidal, trypanocidal, and antitumor [24,27,28].

The unripe dried fruits of *Piper nigrum* L. (Piperaceae, black pepper) are utilized worldwide as aroma and fragrance agents in culinary dishes [2,29]. Ethnomedicinal uses of piper include menstruation disorders, respiratory infections, fever, gastrointestinal complaints, and skin diseases [30]. The specialized metabolites of black pepper comprise essential oils (up to 7%), alkaloids, flavonoids, lignans, tannins, and anthraquinones [31]. Piper extracts and derived compounds were shown to exert a plethora of biological activities, namely, antimicrobial, antioxidant, anticancer, anti-inflammatory, analgesic, hypoglycemic, hypolipidemic, anticonvulsant, and neuroprotective effects [29,31,32]. 

In order to meet the needs of industrial applications, these spices are largely cultivated, with Asia, Latin America, United States, Mediterranean, and Continental Europe countries as being the main suppliers [22]. Hydrodistillation (steam or water distillation) is utilized at the industrial scale for the essential spice oil isolation, since it is a low cost, simple, and environmentally safe method [33]. Therefore, large amounts of spent plant materials are derived as by-products from the industrial production of essential oils. This solid biomass is either discarded as waste and sent to landfill, incinerated, or used as compost in agriculture [34,35]. Additional means of re-utilization of such by-products have been proposed, including as antioxidant additives to biodiesel [36] and alternative fuel in electricity generation [37,38]. 

Literature data on the recovery and valorization of spent plant material from above-mentioned spices are scarce, with only several reports documenting the use of their spent plant materials as a source of chemicals, e.g., isolation of piperine from black pepper [39] and nutrients, namely, fibers, lipids, and carbohydrates from cumin [40]. Still, a large number of phytochemicals, such as phenolic acids, flavonoids, alkaloids with significant biological activities might be recovered and used to obtain high value-added products. Previously, several studies have reported on the recovery of phenolic fractions from spent plant materials of culinary herbs and spices and their pharmacological effects. For example, extracts from spent materials of *Lavandula × intermedia* Emeric ex Loisel. and *Thymus mastichina* (L.) L. were shown to possess significant free radical-scavenging activity, metal chelating, and reducing ability [41]. In addition, Cid-Pérez et al. [42] showed that extracts derived from spent material of Mexican oregano (*Poliomintha longiflora* A.Gray) displayed potent antioxidant and antimicrobial properties.

In this context, the aim of our study was to evaluate the potential valorization of spent plant materials derived after essential oil isolation from five common spices, namely, cinnamon, cumin, clove, laurel, and black pepper. Therefore, after essential oil isolation, the spent plant materials were extracted with solvent of different polarities. The essential oils and extracts were comprehensively characterized by state-of-the-art chromatographic platforms (gas chromatography coupled with mass spectrometry (GC-MS), GC coupled with flame ionization detection (FID), liquid chromatography coupled with high-resolution tandem mass spectrometry (LC-HRMS/MS)). Subsequently, the putative antioxidant effects of the essential oils and spent material derived extracts were assessed by six evaluation methods (1,1’-diphenyl-2-picrylhydrazyl (DPPH) assay, 2,2′-azino-bis(3-ethylbenzothiazoline) 6-sulfonic acid (ABTS) assay, cupric ion reducing antioxidant capacity (CUPRAC) assay, ferric ion reducing antioxidant power (FRAP) assay, ferrous ion chelating ability (MCA) assay, and phosphomolybdenum assay (PBD)). The potential anti-enzymatic activities were evaluated using five *in vitro* methods (acetylcholinesterase (AChE), butyrylcholinesterase (BChE), tyrosinase, amylase and glucosidase inhibition assays).

## 2. Results and Discussion

### 2.1. GC-MS and GC-FID Analysis of Essential Oils Isolated from Different Spices

The highest essential oil yield, as calculated based on dry plant material weight, was determined for cumin, followed by clove, black pepper, cinnamon, and laurel (Table 1). The phytochemical profile of essential oils obtained by hydrodistillation was assessed by GC analysis (Table 2). 

In the case of cinnamon essential oil (**CiEO**), up to 97.16% of the total constituents were identified, with cinnamaldehyde as the main compound (64.20%), followed by cinnamyl acetate (8.74%), *β*-caryophyllene (4.61%), *β*-phellandrene (4.26%), and eugenol (3.47%). Of the total constituents, 97.84% were identified in cumin essential oil (**CuEO**); cuminaldehyde and safranal were the major compounds (30.96% and 29.59%, respectively), alongside *β*-pinene (18.06%) and *γ*-terpinene (12.21%). Eugenol (74.34%) was the main constituent of clove essential oil (**ClEO**), followed by *β*-caryophyllene (17.89%), eugenyl acetate (5.24%), and *α*-humulene (1.24), together making up to 99.31% of the total constituents. Laurel essential oil (**LaEO**) was characterized by the presence of eucalyptol as the major compound (48.05%), followed by *α*-terpinyl acetate (13.36%) and sabinene (7.14%), and other minor constituents, together representing 96.55% of the total volatile fraction. In black pepper essential oil (**BpEO**), 99.17% of the volatile compounds were identified, with *α*-terpinene (24.71%), limonene (19.90%), *β*-caryophyllene (17.59%), and *β*-pinene (13.16%) as the main constituents. The profile of the investigated spice essential oils is well-known, literature data abound with phytochemical studies that document their marker compounds. Indeed, the qualitative and quantitative profile of essential oils varies upon several factors, e.g., plant phenotype, pedoclimatic conditions, methods of harvesting, storage conditions, processing, and extraction methods [43,44]. Our data is in agreement with previous reports on marker constituents from cinnamon—cinnamaldehyde, eugenol, β-caryophyllene, and cinnamyl acetate [7,8,10]; clove—eugenol, eugenyl acetate, *β*-caryophyllene, and *α*-humulene [11,14]; cumin—cuminaldehyde, *β*-pinene, *γ*-terpinene, and safranal [10,43]; laurel—eucalyptol (1,8 cineole), sabinene, and *α*-terpinyl acetate [24]; and black pepper—*β*-caryophyllene, limonene, and *α*-terpinene [31,45].

### 2.2. LC-HRMS/MS Analysis of Extracts Obtained from Different Spices

The spice spent materials obtained after essential oil isolation were extracted with solvents with different polarities (hexane, dichloromethane, methanol, and 50% aqueous methanol) (Table 1) and phytochemically profiled by LC-HRMS/MS (Table 3). For all samples, the highest extraction yields were provided by methanol, reinforcing the well-known ability of this solvent to efficiently solubilize both lipophilic and hydrophilic constituents [46]. 

Twelve specialized metabolites were putatively labeled in the cinnamon extracts, with seven organic and phenolic acids (quinic, gluconic, vanillic, citric, ferulic, caffeoylquinic, and hydroxybenzoic acids), two proanthocyanidins (one monomer, (epi)catechin, and one trimer), and two fatty acids (trihydroxy- and dihydroxy-octadecenoic acids). Previous profiling studies revealed a more complex chemical composition of various cinnamon extracts obtained from unprocessed materials. For instance, Vallverdú-Queralt et al. [47] reported the presence of several phenolic acids (gallic, syringic, hydroxybenzoic, vanillic, chlorogenic, neochlorogenic, cryptochlorogenic, ferulic, rosmarinic, coumaroylquinic, and dicaffeoylquinic acids; *O*-hexosides of homovanillic, caffeic, and coumaric acids, proanthocyanidins (catechin, epicatechin, proanthocyanidin trimers and hexamers) and flavonoids (free aglycons and *O-* or *C*-glycosides of quercetin, kaempferol, naringenin and hesperitin) in a 50% aqueous ethanol cinnamon extract. In another study, 28 specialized metabolites (hydroxybenzoic acids, hydroxycinnamic acids, hydroxyphenylacetic acids, flavanols, flavones, flavonols, isoflavonoids, curcuminoids, tyrosols, and lignans) were tentatively identified in a 70% aqueous ethanol cinnamon extract [48]. 

With respect to the cumin extracts, eleven constituents were annotated; some of them (citric acid, coniferol-*O*-hexoside) were already noticed in the cinnamon extracts. In contrast, the flavonoid profile was slightly more complex, with seven luteolin and apigenin derivatives, such as free aglycons, *O*-hexosides, acetylhexosides or hexuronide-hexosides; additionally, two hydroxycinnamic acid derivatives were observed (Table 3). Previously, Ali et al. [48] reported around 24 constituents in a 70% aqueous ethanol cumin extract, whereas Vallverdú-Queralt et al. [47] described 34 different chemical entities in the 50% aqueous ethanol cumin extract; the most notable compounds belonged to the phenolic acids and flavonoids (flavanols, flavones, flavonols), which is in agreement with the results from the current study. 

The LC-HRMS/MS analysis of clove extracts revealed the presence of 19 constituents; beside the common organic acid derivatives, such as quinic, gallic, and citric acids, several specific metabolites were tentatively labeled (Table 3). For instance, hydroxycinnamic acids and flavonoids were represented by twelve derivatives (caffeoylquinic acids I and II, quercetin, isorhamnetin and their *O*-glycosides, luteolin, chrysoeriol, cirsiliol, cirsimaritin). On the other hand, hydrolysable tannins, namely, ellagic acid, trimethylellagic acid, di- and tri-*O*-galloyl-hexahydroxydiphenoyl-hexoses, were retrieved as distinguishable clove constituents. Previously, Ali et al. [48] documented around 20 different phenolic acids and flavonoids in the 70% aqueous ethanol clove extract. Duyen Vu et al. [49] reported the characterization of 28 compounds (11 flavonoids, 13 triterpene acids, and four phenolic acids) in a 70% aqueous ethanol clove extract of *Syzygium formosum* (Wall.) Masam, whereas Peixeto Araujo et al. [50] described 153 phytochemicals from different classes, including organic acids, phenolic acids, flavonoids, in the leaves, pulp and seed extracts of *Eugenia calycina* Cambess. Rummun et al. [51] revealed the presence of 59 metabolites, which consisted mostly of phenolic acids, flavonoids, and hydrolysable tannins, in the leaf extracts of *Syzygium coriaceum* Bosser & J. Gueho. 

Except for citric acid, caffeoylquinic acid, gallic acid-*O*-hexoside, and a proanthocyanidin trimer, the remaining 11 compounds tentatively identified in the laurel extracts were derivatives of quercetin, isorhamnetin, and kaempferol (Table 3). Our data is in agreement with the study of Pacifico et al. [52] who documented the presence of 21 diverse flavonoids and proanthocyanidins in the methanolic laurel leaf extracts and that of Bourebaba et al. [53] in which 15 phenolic acids and flavonoids were labeled in the ethanol laurel leaf extracts. It is worth mentioning that three acylated kaempferol glycosides (**La11-La13**) were previously reported only by Fiorini et al. [54] in a 70% aqueous ethanol extract of laurel leaves. 

Lastly, the LC-HRMS/MS metabolite profiling of the black pepper extracts revealed a totally different chemical composition, as compared to other four spices, with 26 out of a total of 30 tentatively labeled compounds represented by piperamides (Table 3). Among them, piperine, piperlonguminine, pellitorine, pipernonaline, retrofractamide B, guineensine, and *N*-isobutyl-dodecadienamide are some of the most notable congeners. A similar chemical composition was documented in previous literature data for different black pepper extracts [55,56,57]. 

To sum up, the LC-HRMS/MS analysis of the extracts obtained with solvents with different polarities from the plant materials after hydrodistillation (usually discarded as waste products by the essential oil industry) revealed the presence of a diverse range of bioactive phytochemicals. Nevertheless, the adequate extraction solvent should be selected considering the category of phytochemicals as well as the bioactivities that are targeted; for instance, our data (Table 3) showed that cinnamon, cumin, clove, and laurel contained numerous polar flavonoids and phenolics, which were present almost exclusively in the methanol and 50% aqueous methanol extracts, whereas black pepper was rich in non-polar piperamides, easily extractible with hexane, dichloromethane, or methanol. Overall, as compared to previously literature data, the LC- HRMS/MS analysis revealed a lesser phenolic profile of the investigated spices; such differences could be due not only to the extraction conditions (processed/spent vs. unprocessed material), but also to plant phenotype, pedoclimatic conditions, harvesting, and storage conditions.

### 2.3. Total Phenolic and Flavonoid Content of Extracts Obtained from Different Spices

The spice residue extracts obtained after the essential oil isolation were next evaluated with respect to their total phenolic content (TPC) and total flavonoid content (TFC) (Table 4). In the cinnamon extracts, TPC varied between 14.00 mg gallic acid equivalents (GAE)/g extract (**CiD**) and 92.90 mg GAE/g (**CiMW**), whereas TFC ranged from 1.76 mg rutin equivalents (RE)/g extract (**CiMW**) and 9.83 mg RE/g [58]. According to the existing literature data, total phenolics levels retrieved in our samples are significantly lower than those previously reported in the 50% aqueous acetone, 80% aqueous methanol, or aqueous cinnamon extracts (148–309 mg GAE/g) [59,60]. Cumin extracts displayed TPC from 7.86 (**CuH**) to 30.77 mg GAE/g (**CuMW**), with a significantly higher TFC, especially for the polar extracts (50.68 mg RE/g in **CuM** and 66.25 mg RE/g in **CuMW**); these two extracts also showed the highest flavonoid levels from all analyzed spices (Table 4). A similar trend was previously observed by Gupta [61] with a reported TPC of 12.15 mg GAE/g and 46.56 mg quercetin equivalents/g in a methanol cumin extract. On the other hand, Rebey et al. [62] showed TPC and TFC between 1.09 and 18.60 mg GAE/g and 0.52–5.91 mg catechin equivalents/g, respectively, in cumin extracts obtained with solvents with different polarities (ethanol, methanol, acetone, water, 80% acetone, 80% methanol, 80% ethanol); generally, the hydro-organic extracts showed superior TPC and TFC, which is in agreement with the results from our study. 

All four clove extracts displayed the highest TPC (90.67–103.76 mg GAE/g) of all analyzed spices; in contrast, TFC was significantly lower, especially in the non-polar extracts (1.43 mg RE/g in **ClH** and 5.89 mg RE/g in **ClD**), suggesting that the contribution to TPC values is not due to the presence of flavonoids. Previous reports showed even higher TPC for various aqueous, ethanol, or methanol clove extracts (212.85–425.04 mg GAE/g) [59,61], whereas the levels of flavonoids (4.70–17.5 mg quercetin equivalents/g) in polar clove extracts [63] were significantly lower than the one reported in the current study (41.71 mg RE/g in **ClMW** and 31.00 mg RE/g in **ClM**). With respect to the laurel extracts, TPC varied from 22.49 (**LaH**) to 75.53 mg GAE/g (**LaMW**), whilst TFC varied from 0.50 (**LaH**) to 27.33 mg RE/g (**LaM**). Previous data revealed a high variability of the phenolics profile, with TPC ranging from 30.73 to 135.47 mg GAE/g for laurel extracts obtained with polar solvents (methanol/ethanol-water) under different extraction conditions (heat-reflux, ultrasound-assisted, microwave-assisted, mechanochemical-assisted) [64,65]. Lastly, black pepper extracts showed a homogenous TPC trend, with amounts between 31.50 (**BpH**) and 37.01 mg GAE/g (**BbMW**); a significantly lower TFC, ranging from 2.31 (**BpMW**) and 6.97 mg RE/g (**BpM**), was noticed. These results are in agreement with previous literature reports; for instance, different black pepper methanolic extracts displayed total phenolics and flavonoids levels between 36.71 and 58.90 mg GAE/g and 5.42 and 18.37 mg RE/g, respectively [56,66]. 

Overall, our data suggest that the spent plant materials after essential oil isolation can be confidently used for the efficient recovery of phenolics and flavonoids; a high extractability of these categories of phytochemicals could be a good indicator for a wide range of biological activities with putative applications in the management of diverse chronic diseases. 

### 2.4. Antioxidant Activity of Extracts and Essential Oils Obtained from Different Spices

The antioxidant properties of spices are largely known; besides their common use as food preservatives, spices display additional health benefits in the prevention and complementary treatment of oxidative stress-related disorders (e.g., cardiovascular diseases, neurodegenerative diseases, gastro-intestinal disorders, and cancers) [1,67]. Polyphenols and terpenoids are considered the main antioxidant compounds from spices that act as radical scavenging, reducing power, and metal chelating agents [3]. To evaluate the antioxidant capacity of the essential oils and their spent plant material extracts, several *in vitro* methods were undertaken, namely, 1,1’-diphenyl-2-picrylhydrazyl (DPPH) assay, 2,2′-azino-bis(3-ethylbenzothiazoline) 6-sulfonic acid (ABTS) assay, cupric ion reducing antioxidant capacity (CUPRAC) assay, ferric ion reducing antioxidant power (FRAP) assay, ferrous ion chelating ability (MCA) assay, and phosphomolybdenum assay (PBD). The obtained results are summarized in Table 5. By using an integrative approach, we report herein for the first time the antioxidant effects of spent spice material extracts derived after essential oil hydrodistillation from cinnamon, cumin, clove, laurel, and black pepper. In order to illustrate that the spent spice material is a valuable source of phytochemicals, we compared our results with literature data on unprocessed plant material. Still, even though several studies assessed the antioxidant potential of investigated spices, a direct comparison between the results obtained in the present study and literature data is hampered as a consequence of the implementation of different protocols and various ways of expressing the results.

All investigated extracts displayed variable radical scavenging abilities, as assessed by the DPPH and ABTS assays. Thus, cinnamon extracts showed significant radical scavenging effects, with **CiMW** exhibiting the highest activity in the DPPH test and **CiEO** in the ABTS assay (210.00 and 839.94 mg Trolox equivalents (TE)/g, respectively). The polar extracts (**CiMW** and **CiM**) were the most effective as scavenging agents, while the non-polar extracts (**CiH** and **CiD**) were significantly less active (Table 5). A similar trend was observed by Saranya et al. [68], when the anti-DPPH radical effects of cinnamon extracts decreased in the following order: methanol (IC_50_ = 11.9 µg/mL) > chloroform (IC_50_ = 166.3 µg/mL) > hexane (IC_50_ = 689.2 µg/mL). Regarding the cumin extracts, **CuMW** displayed the highest DPPH and ABTS radical scavenging potency, with values of 46.39 and 77.14 mg TE/g, respectively. The non-polar extracts were less potent, while **CuEO** showed no activity in the DPPH assay. All clove extracts were significantly effective as radical scavengers, with **ClEO** exhibiting the highest activity towards DPPH and ABTS radicals among investigated spices (values of 525.78 and 936.44 mg TE/g, respectively) (Table 5). In addition, the spent material extracts were shown to exhibit anti-radical effects, with values ranging from 355.23 (**ClD**) to 488.45 mg TE/g (**ClMW**) in the DPPH test, and from 669.32 (**ClD**) to 770.12 mg TE/g (**ClMW**) in the ABTS assay. Previously, clove essential oil was reported to scavenge DPPH and ABTS radicals in a similar extent (EC_50_ values of 0.40 and 0.42 mg/mL, respectively) to an ethanol extract (EC_50_ values of 0.41 and 0.43 mg/mL, respectively) [69]. The DPPH scavenging ability of laurel extracts varied between 2.08 mg TE/g (**LaH**) and 194.14 mg TE/g (**LaMW**), while **LaEO** exhibited the highest activity in the ABTS assay (507.33 mg TE/g). Speroni et al. [70] documented the radical quenching capacity of methanol and chloroform laurel extracts (DPPH: IC_50_ of 38 and 155 µg/mL; ABTS: 0.65 and 0.21 mmol TE/g). Within the black pepper extracts, the polar extracts **BpMW** and **BpM** were the most effective in scavenging synthetic radicals (DPPH: 45.41 and 32.41 mg TE/g; ABTS: 76.59 and 49.88 mg TE/g), followed by the non-polar extracts, **BpH** and **BpD**. In contrast, **BpEO** was less active compared to other extracts in the two assays (Table 5). In our previous study, we reported a similar radical quenching ability for the methanol extracts derived from *Piper* sp. (63.67–82.44 mg TE/g in DPPH assay and 61.63–77.60 mg TE/g in ABTS assay) [56].

The reducing potential, as assessed by CUPRAC and FRAP methods, varied largely between the investigated extracts. In the case of cinnamon extracts, their ability to reduce transition metals displayed a similar pattern and decreased in the following order: **CiEO** > **CiMW** > **CiM** > **CiD** > **CiH**, with the highest potential at 762.89 mg TE/g and 834.77 mg TE/g in the CUPRAC and FRAP assays, respectively. Moreover, the polar extracts showed greater electron donor capacity as compared to the non-polar extracts (Table 5). Similarly, previous reports on cinnamon extracts demonstrated their power reducing ability, with methanol extracts being more potent ferric ion reducers (0.74 molar ascorbic acid equivalents (MAAE)/g) when compared to non-polar solvent extractives, e.g., hexane (0.28 MAAE/g) [68]. Among the cumin extracts, the most effective reducing agents were found to be **CuMW** (118.66 mg TE/g) and **CuEO** (87.71 mg TE/g) in the CUPRAC and FRAP assays, respectively. The reducing potential of cumin essential oil and methanol extract were previously assessed by Einafshar et al. [71] using the FRAP method (459.4 and 18.47 mmol Fe^2+^/g, respectively). Clove extracts displayed a noticeable reducing power, with **ClEO** being the most active among all investigated spices (2848.28 mg TE/g and 1927.98 mg TE/g in the CUPRAC and FRAP assays, respectively). Nonetheless, the spent spice material extracts exhibited significant reducing potential, that varied between 753.39 (**ClD**) and 978.08 mg TE/g (**ClMW**) in the CUPRAC assay, and from 876.30 (**ClM**) to 1209.92 mg TE/g (**ClH**) in the FRAP assay. Compared to clove, laurel extracts were less active as transition metal reductants (Table 5). Their reducing power varied with a similar pattern in both assays, increasing from 28.58 (**LaH**) to 416.67 mg TE/g (**LaEO**) in the CUPRAC test, and between 14.11 (**LaH**) and 435.97 mg TE/g (**LaEO**) in the FRAP test. Besides, the polar fractions were more active compared to non-polar extracts, displaying up to a 10-fold and 14-fold activity increase in the CUPRAC and FRAP assays, respectively (Table 5). In accordance to our study, Carlsen et al. [72] reported superior reducing effects for clove compared to laurel 50% methanol extracts as evaluated by FRAP method (273.3 vs. 27.8 mmol TE/100 g). The reducing properties of black pepper extracts varied from 51.22 (**BpM**) to 176.06 mg TE/g (**BpEO**) in the CUPRAC assay, while in the FRAP assay activity values from 14.11 (**LaH**) to 435.97 mg TE/g (**LaEO**) were noticed. In our previous study on *Piper* species, the methanolic extracts were shown to be active in both CUPRAC and FRAP assays, their reducing capacity varying from 37.36 to 140.52 mg TE/g, and from 16.05 to 77.00 mg TE/g, respectively [56]. Moreover, a comprehensive report on antioxidant effects of 39 spices revealed the reducing potency (as evaluated by CUPRAC method) of methanol extracts obtained from cinnamon (53.65 mg TE/g), cumin (1.71 mg TE/g), clove (54.47 mg TE/g), laurel (11.34 mg TE/g), and black pepper (2.09 mg TE/g) [73].

With respect to metal chelation capacity, the analyzed extracts showed variable degrees of potency, with **LaEO** displaying the highest potency (33.78 mg EDTAE/g), followed by **BpMW** (30.51 mg EDTAE/g), **CuMW** (26.96 mg EDTAE/g), **ClMW** (22.78 mg EDTAE/g), and lastly, **CiH** (20.46 mg EDTAE/g)**.** It was shown that the residual water derived from laurel essential oil distillation possesses notable chelating ability (16 mg EDTAE/g). Using similar experimental settings, Luca et al. [56] found that black pepper methanolic extracts acted as iron chelators (12.35–21.54 mg EDTAE/g). Hydroalcoholic cumin extracts were reported to have iron-chelating effects, with IC_50_ values ranging from 29.35 to 35.46 mg/mL [74]. Gülçin et al. [75] found that water and ethanol clove extracts at 60 µg/mL displayed a chelating capacity of 84% and 88%, respectively. 

Regarding the total antioxidant capacity evaluated by the PBD assay, the essential oils displayed the highest potential, with values from 18.26 (**CiEO**) to 68.19 mmol TE/g (**ClEO**). Concerning the spent spice material extracts, the obtained values were similar to each other within the same species. Thus, clove extracts were prompted as being the most active in the PBD test, their total antioxidant activity varying from 4.38 (**ClM**) to 6.02 mmol TE/g (**ClH**). Cinnamon and laurel extracts also showed good antioxidant capacity in the PBD assay (1.22–2.14 mmol TE/g and 1.99–2.07 mmol TE/g, respectively). According to recent literature data, a cinnamon methanol extract was shown to exhibit a total antioxidant ability of 149.15 mg GAE/g [76]. Albayrak et al. [77] studied the total antioxidant activity of laurel extracts and found that it decreased in the following order: methanol (196.98 mg ascorbic acid equivalents (AAE)/g) > infusion (69.50 mg AAE/g) > decoction (35.65 mg AAE/g). Contrarily, cumin and black pepper extracts exhibited the lowest potencies among all spices, with activity values below 1.35 mmol TE/g (Table 5). Previously, the total antioxidant potency of hydroalcoholic cumin extracts varied between 8.25–11.24 mg/mL [78]. In one of our previous studies, we showed that a black pepper methanol extract had a total antioxidant capacity of 1.24 mmol TE/g [56].

**Figure 1:**
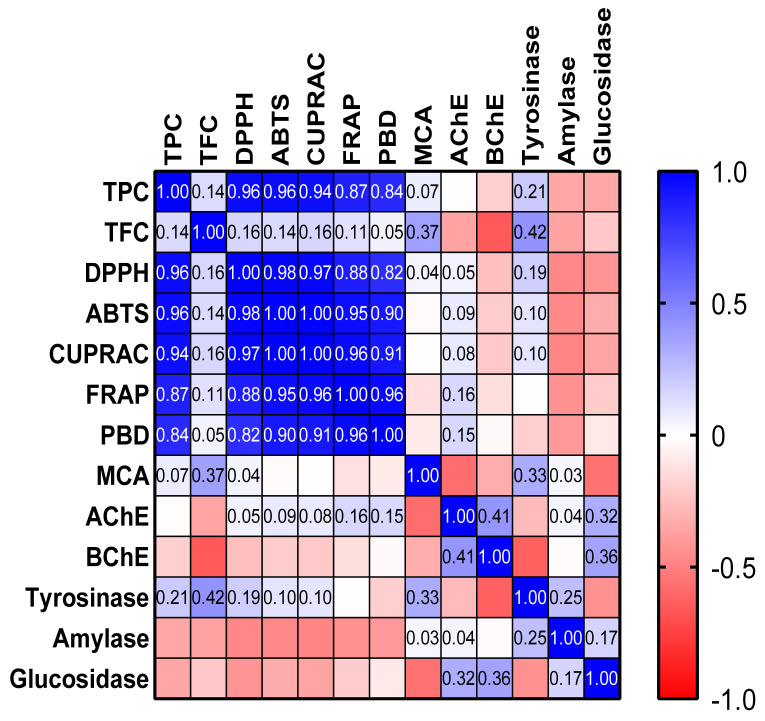
Pearson’s correlation values (*r*) in the performed biological activity assays; AChE—acetylcholinesterase; BChE—butyrylcholinesterase; CUPRAC—cupric ion reducing antioxidant capacity; DPPH—1,1-diphenyl-2-picrylhydrazyl; FRAP—ferric ion reducing antioxidant power; MCA—metal chelating activity; PBD—phosphomolybdenum assay; TFC—total flavonoid content; TPC—total phenolic content.

To summarize, the spent plant material extracts of the studied spices exhibited significant antioxidant properties, with different degrees of activity. It is noteworthy to mention the remarkable antioxidant effects of cinnamon and clove extracts, as revealed by the radical scavenging capacity and reducing power assays (Table 5). In accordance with our study, Assefa et al. [73] highlighted the clove and cinnamon extracts among 39 common spices as notable sources of antioxidants able to prevent the deleterious effects induced by oxidative stress. As depicted in Figure 1, significant correlations between TPC and free radical scavenging activity, reducing power, and total antioxidant capacity were established (*r* > 0.8). Similarly, several studies have demonstrated a significant correlation between antioxidant properties of spices and their total amount of phenolic compounds [47,73,79]. No relevant correlation was found between TFC and the observed antioxidant effects (*r* < 0.37). Therefore, we can assume that phenolic compounds are the main contributors to the overall antioxidant activity of investigated extracts. In addition, TPC and TFC did not correlate with metal-chelating activity, thus other constituents might be responsible for the chelation capacity of the spent spice materials.

### 2.5. Anti-Enzymatic Activity of Extracts and Essential Oils Obtained from Different Spices

For a better overview on the potential involvement of cinnamon, cumin, clove, laurel, and black pepper in the management of different chronic diseases, we next investigated the *in vitro* anti-enzymatic activity of the essential oils as well as of their spent spice material extracts (Table 6). As target enzymes, AChE, BChE, tyrosinase, amylase, and glucosidase were selected, since they are considered to be directly linked to the etiopathogeny of Alzheimer’s disease, skin hyperpigmentation, or type II diabetes mellitus [56]. To the best of our knowledge, there are no previous comprehensive studies assessing the multi-enzymatic properties of these five spices. Similar to the antioxidant assays, we compared our results with literature data on unprocessed plant material.

The anti-AChE effects of cinnamon extracts varied between 1.14 mg galanthamine equivalents (GALAE)/g (**CiD**) and 2.01 mg GALAE/g [58], whereas slightly better anti-BChE properties were noticed, especially for **CiM** extract (Table 6). Interestingly, **CiEO** was somehow active against AChE, but inactive against BChE, which could indicate a potential selectivity against the two enzymes. Previously, an ethanol cinnamon extract (200 μg/mL) was shown to exhibit 54.30% and 66.43% inhibition against AChE and BChE, respectively [80]. Regarding cumin extracts, the non-polar extracts (**CuH** and **CuD**) showed the highest AChE and BChE inhibitory activities, with the other three samples being inactive, especially against BChE. Furthermore, **CuH** displayed the highest anti-AChE effects from all tested spices (2.95 mg GALAE/g). Mahnashi et al. [81] showed considerable inhibitory activities against AChE and BChE with IC_50_ values of 198 and 37 μg/mL, respectively. The anti-cholinesterase activity of clove extracts varied in the following order: **ClEO** > **ClH** > **ClD** > **ClM** > **ClMW,** with the highest inhibitory capacity at 2.77 and 2.97 mg GALAE/g in AChE and BChE assays, respectively (Table 6). In a previous study, IC_50_ values of 49.73–61.50 and 88.14–103.53 μg/mL were reported for a clove methanol extract and essential oil in AChE and BChE assays, respectively [82]. The non-polar **LaH** extract exhibited the highest anti-BChE effects from all analyzed spices (3.41 mg GALAE/g); in contrast, **LaEO** displayed very low BChE inhibitory properties (0.41 mg GALAE/g). With respect to the anti-AChE activity, the effects varied from 1.04 (**LaMW**) to 2.47 mg GALAE/g (**LaH**). Ferreira et al. [83] showed that the laurel essential oil, aqueous, and ethanol extracts produced AChE inhibitions of 51.3% 19.9%, and 48.4%, respectively, at 0.5 mg/mL; there was no information about their BChE inhibitory properties. Lastly, **BpD** exhibited the highest anti-AChE ability (2.55 mg GALAE/g), whereas the highest anti-BChE effects (2.46 mg GALAE/g) were noticed for **BpMW**. In a previous study, Luca et al. [56] showed that the AChE and BChE inhibitory activity of various pepper samples varied from 0 to 2.35 mg GALAE/g and 0.60 to 3.11 mg GALAE/g, respectively.

When assessing tyrosinase inhibition (Table 6), it was noticed that the polar **CiMW** and **CiM** extracts clearly displayed the most potent effects (66.45 and 61.56 mg kojic acid equivalents (KAE)/g, respectively), which were 2.5–3.6-fold higher than the remaining cinnamon samples. Previously, Tamfu et al. [80] showed that an ethanol cinnamon extract (200 μg/mL) exhibited a 45.37% inhibition against tyrosinase. Cumin extracts exerted a comparable anti-tyrosinase activity, with the highest effects also noticed in the polar extracts **CuM** (58.05 mg KAE/g) and **CuMW** (63.12 mg KAE/g). Mukherjee et al. [84] and Gholamhoseinian and Razmi [85] reported tyrosinase inhibitions of 52% and 64.17% for methanol cumin extracts at 1.14 and 1 mg/mL, respectively. The tyrosinase inhibitory properties of clove extracts varied between 20.79 (**ClH**) and 59.23 mg KAE/g (**ClMW**), with **ClEO** showing an inhibition of only 38.16 mg KAE/g. The anti-tyrosinase abilities of clove have been scarcely reported; for instance, Ahmed et al. [86] compared the activity of different clove extracts; with respect to the IC_50_ values, the following order was observed: essential oil (12.1 μg/mL) > hexane extract (17.4 μg/mL) > methanol extract (30.1 μg/mL) > ethanol extract (65.3 μg/mL). Within the series of laurel extracts, **LaEO**, **LaM**, and **LaMW** showed anti-tyrosinase effects to different extents (22.48–43.38 mg KAE/g), whereas the non-polar extracts were inactive. Deniz et al. [87] noticed that the 80% ethanol extracts of laurel exhibited a low tyrosinase inhibition (34.09% at 666 μg/mL). With respect to the black pepper extracts, it was observed that the non-polar extracts (**BpH** and **BpD**) had the most potent anti-tyrosinase abilities; in contrast, **BpEO** was the weakest tyrosinase inhibitor (Table 6). Previously, it was reported that black pepper extracts were able to significantly down-regulate mushroom tyrosinase (monophenolase and diphenolase) as well as murine tyrosinase [56]. 

Regarding the amylase and glucosidase inhibition, the five spice extracts displayed weak inhibitory properties; generally, they exhibited slightly better anti-glucosidase effects than anti-amylase effects. From all tested samples, **LaEO** presented the most notable amylase inhibition (1.29 mg acarbose equivalents (ACAE)/g) followed by **BpH** and **BpD** (1.00 and 0.99 mg ACAE/g, respectively); **CiEO**, **CuEO**, and **BpEO** were inactive against the same enzyme. From all essential oils, only **ClEO** was able to inhibit glucosidase (1.00 mg ACAE/g); nonetheless, the spice residue extracts showed lower anti-glucosidase effects (max. 0.88–0.89 mg ACAE/g in **CiH**, **CiD**, **ClM**, and **LaM**) (Table 6). The modulation of the two anti-diabetic enzymes by different spices was found to be in agreement with our data; for example, several cinnamon aqueous extracts were shown to inhibit amylase, maltase, and sucrose, with IC_50_ values of 1.23–1.77, 0.58–1.96, and 0.42–2.96 mg/mL, respectively [88], whereas a cumin ethanol extract displayed inhibitory effects against glucosidase of 45% at 100 μg/mL [89]. An 80% aqueous acetone clove extract was reported to exhibit anti-glucoside and anti-amylase effects, with IC_50_ values of 145.07 and 497.27 μg/mL, respectively [90], whilst the clove essential oil displayed IC_50_ values of 71.94 and 88.89 μg/mL against the same two enzymes [91]. Moreover, a laurel extract exerted a 47.26% inhibition against glucosidase at 200 μg/mL [92], whereas different black pepper extracts showed weak anti-glucosidase effects of 0.84–1.22 mmol ACAE/g [56]. 

Overall, no significant correlations between the AChE, BChE, amylase, and glucosidase inhibitory activity of the investigated spices and their TPC and TFC were noticed. On the one hand, this can suggest that other categories of compounds might contribute to the observed effects; on the other hand, the tested spices exhibited modest anti-enzymatic activities, making the unveiling of the responsible biomolecules difficult. Nevertheless, the most promising anti-enzymatic effects were noticed with respect to tyrosinase; in addition, weak and moderate correlations between TPC (*r =* 0.21) and TFC (*r =* 0.42), respectively, and anti-tyrosinase activity were observed (Figure 1).

## 3. Material and Methods

### 3.1. Plant Materials

Dried plant materials (cinnamon barks, cumin fruits, clove buds, laurel leaves, and black pepper fruits) were purchased from pharmacies and local markets and their botanical identity was confirmed by one of the authors (A.T.). Voucher specimens (Ci 1/2020, Cu 2/2020, Cl 3/2020, La 4/2020, Bp 5/2020) were deposited in the Department of Pharmacognosy, Faculty of Pharmacy, “Grigore T. Popa” University of Medicine and Pharmacy Iasi, Romania.

### 3.2. Extraction

#### 3.2.1. Essential Oil Isolation

The powdered plant materials (50 g each) were subjected to hydrodistillation for 3 h in a Clevenger-type apparatus. EOs were dried over anhydrous sodium sulfate and stored in dark glass tubes at a temperature of 4 °C until further analysis.

#### 3.2.2. Preparation of Spent Plant Material Extracts

The spent plant materials (after essential oil isolation) were dried at 40 °C (48 h) and further extracted using solvents of different polarities (hexane, dichloromethane, methanol, and 50% aqueous methanol) by ultra-sonication (3 cycles of 30 min each, at room temperature). The obtained extracts were evaporated to dryness under vacuum (with the yields provided in Table 1) and kept at −20 °C until subsequent analysis. 

### 3.3. GC-MS and GC-FID Analysis

An Agilent 6890N gas chromatograph [93] coupled with a mass spectrometer (MS) detector (Agilent model 5975 inert XL) and a flame ionization detector (FID) was used to assess the qualitative and quantitative profile of essential oils isolated from investigated spices. TRB-5MS capillary columns (30 m length, 0.25 mm internal diameter, 0.25 μm film thickness) were used for each detector. The analysis was conducted according to a previously described method [94]. Retention indices were calculated for the individual compounds using a standard mixture of C8–C20 *n*-alkanes. The compounds from the essential oils were identified by comparison of their mass spectra with those from the NIST 11 Mass Spectra Library, and their retention indices with literature data (NIST Chemistry WebBook; [94,95]). The relative percentages of individual compounds were obtained from the FID peak areas without using correction factors. 

### 3.4. LC-HRMS/MS Analysis

The LC-HRMS/MS was performed on an Agilent 1200 HPLC system (Agilent Technologies, Palo Alto, CA, USA) equipped with a binary pump (G1312C), column thermostat (G1316A), auto-sampler (G1329B), and accurate-mass quadrupole-time-of-flight MS detector (G6530B). The separations were carried out on a Phenomenex Gemini C18 column (2 × 100 mm, 3 μm), with phase A (0.1% formic acid in water) and phase B (0.1% formic acid in acetonitrile); the mobile phase gradient was as follows: 10–60% B (0–45 min) and 90% B (46–50 min); flow rate 0.2 mL/min; injection volume 2 μL. The following MS parameters were set: electrospray ionization source (ESI); full-scan high-resolution accurate-mass acquisition mode; negative and positive ionization modes; *m/z* range 50–1000; N_2_ flow rate 12 L/min, vaporizer temperature 350 °C, nebulizer pressure 40 psi, capillary voltage 4000 V, skimmer 65 V, fragmentor 140 V, and collision-induced dissociation (CID) energy 40 V. Data acquisition and analysis were achieved with MassHunter Workstation Data Acquisition 8.0 and Qualitative Navigator 8.0, respectively. The assignment of the peaks observed in the base peak chromatograms (BPC) of the different spice extracts was performed by comparing the spectrometric data with previous literature data reporting on the LC-MS analysis of similar constituents or online databases (METLIN, KNApSacK, PubChem, NIST Chemistry WebBook). 

### 3.5. TPC, TFC, Antioxidant and Anti-Enzymatic Assays

TPC and TFC were determined according to previously described methods [96,97] and expressed as mg GAE/g (TPC) and mg RE/g (TFC). DPPH, ABTS, CUPRAC, and FRAP were performed as in [96,97], with the results presented as mg TE/g. MCA and PBD were carried out as described in [96,97], with the data provided as mg EDTAE/g (MCA) and mmol TE/g (PBD). AChE, BChE, tyrosinase, amylase, and glucosidase inhibition methods were detailed in [96,97]. The anti-enzymatic activities were expressed as mg GALAE/g in AChE and BChE assays, mg KAE/g d.w. in tyrosinase assay and mmol ACAE/g d.w. in amylase and glucosidase assays. 

### 3.6. Statistical Analysis

All the experiments were performed in three replicates, with the results presented as mean ± standard deviation (SD). One-way analysis of variance (ANOVA) with Turkey’s post hoc test was conducted; *p* < 0.05 was considered statistically significant. The correlation analysis between TPC, TFC, and biological activities was reported as Pearson’s coefficients, calculated using R software (v. 3.6.2).

## 4. Conclusions

The GC-MS and GC-FID analyses of the essential oils obtained from five common spices pointed out several phytochemical markers: cinnamaldehyde in cinnamon; cuminaldehyde and safranal in cumin; eugenol in clove; eucalyptol in laurel; and *α*-terpinene, limonene, *β*-caryophyllene, and *β*-pinene in black pepper. On the other hand, the LC-HRMS/MS profiling of the spent plant materials extracted with different polarity solvents revealed a complex phytochemical composition of the same spices: organic acids, phenolic acids, proanthocyanidins, and oxygenated fatty acids in cinnamon; flavonoids (free aglycons or glycosides of luteolin and apigenin) in cumin; flavonoids (free aglycons or glycosides of quercetin, isorhamnetin, luteolin, chrysoeriol, cirsiliol, cirsimaritin) and hydrolysable tannins in clove; proanthocyanidins and flavonoids (free aglycons and glycosides of quercetin, isorhamnetin and kaempferol) in laurel; and piperamides (piperine, piperlonguminine, pellitorine, pipernonaline, retrofractamide B, guineensine and *N*-isobutyl-dodecadienamide) in black pepper. 

The comparative assessment of the antioxidant potential of the essential oils and spent spice material extracts indicated a very strong activity for all samples, as depicted from the significantly high values obtained in radical scavenging (ABTS, DPPH), metal chelating (MCA), reducing (CUPRAC, FRAP), and PBD tests. Interestingly, significant correlations between TPC and antioxidant activity of spent extracts were observed, with no relevant correlations between their TFC and the antioxidant effects. Lastly, the anti-enzymatic assays unveiled that the investigated spices exhibited a mild modulation of several key enzymes (cholinesterases, tyrosinase, glucosidase, and amylase) targeted in the management of chronic diseases, such as Alzheimer’s disease, type 2 diabetes mellitus, or skin disorders.

Overall, our study shows that, not only the volatile fractions of common spices, such as cinnamon, cumin, clove, laurel, and black pepper, but also their spent plant materials remained after hydrodistillation, and often discarded by the essential oil industry, can be further considered as rich sources of exploitable bioactive molecules endowed with antioxidant and multi-enzymatic inhibitory activities. 

## Figures and Tables

**Table 1:** Extraction yields of essential oils and extracts obtained from different spices.

Sample	Extraction Yield
	(%)
**Cinnamon Extracts**	
**CiH**	0.29
**CiD**	0.55
**CiMW**	1.71
**CiM**	1.77
**CiEO**	3.10
**Cumin Extracts**	
**CuH**	2.53
**CuD**	3.30
**CuMW**	1.94
**CuM**	3.69
**CuEO**	7.40
**Clove Extracts**	
**ClH**	8.98
**ClD**	14.37
**ClMW**	1.36
**ClM**	27.85
**ClEO**	7.00
**Laurel Extracts**	
**LaH**	3.23
**LaD**	4.89
**LaMW**	4.82
**LaM**	9.03
**LaEO**	3.00
**Black Pepper Extracts**	
**BpH**	1.39
**BpD**	4.44
**BpMW**	2.85
**BpM**	8.51
**BpEO**	5.80

Abbreviations: BpD—black pepper dichloromethane extract; BpEO—black pepper essential oil; BpH—black pepper hexane extract; BpM—black pepper methanol extract; BpMW—black pepper 50% aqueous methanol extract; CiD—cinnamon dichloromethane extract; CiEO—cinnamon essential oil; CiH—cinnamon hexane extract; CiM—cinnamon methanol extract; CiMW—cinnamon 50% aqueous methanol extract; ClD—clove dichloromethane extract; ClEO—clove essential oil; ClH—clove hexane extract; ClM—clove methanol extract; ClMW—clove 50% aqueous methanol extract; CuD—cumin dichloromethane extract; CuEO—cumin essential oil; CuH—cumin hexane extract; CuM—cumin methanol extract; CuMW—cumin 50% aqueous methanol extract; LaD—laurel dichloromethane extract; LaEO—laurel essential oil; LaH—laurel hexane extract; LaM—laurel methanol extract; LaMW—laurel 50% aqueous methanol extract.

**Table 2:** Chemical composition of essential oils isolated from different spices.

No.	RI *	Compound	(%)
	Cinnamon Essential Oil		
1.	937	*α*-pinene	1.05
2.	1008	*α*-phellandrene	1.79
3.	1019	*α*-terpinene	0.92
4.	1027	*m*-cymene	1.31
5.	1039	*β*-phellandrene	4.26
6.	1102	Linalool	2.83
7.	1293	Cinnamaldehyde	64.20
8.	1368	Eugenol	3.47
9.	1433	*β*-caryophyllene	4.61
10.	1457	Cinnamyl acetate	8.74
11.	1465	*α*-humulene	0.82
12.	1535	Methoxycinnamaldehyde	0.60
13.	1775	Benzyl benzoate	2.56
		**Total**	**97.16**
	**Cumin Essential Oil**		
1.	937	*α*-pinene	0.99
2.	976	Sabinene	0.92
3.	984	*β*-pinene	18.06
4.	992	Myrcene	1.08
5.	1028	*m*-cymene	2.67
6.	1066	*γ*-Terpinene	12.21
7.	1180	Terpinen-4-ol	1.39
8.	1255	Cuminaldehyde	30.93
9.	1304	Safranal	29.59
		**Total**	**97.84**
	**Clove Essential Oil**		
1.	1369	Eugenol	74.34
2.	1439	*β*-caryophyllene	17.89
3.	1466	*α*-humulene	1.84
4.	1535	Eugenyl acetate	5.24
		**Total**	**99.31**
	**Laurel Essential Oil**		
1.	938	*α*-pinene	6.29
2.	952	Camphene	0.97
3.	979	Sabinene	7.14
4.	984	*β*-Pinene	4.95
5.	1037	Eucalyptol	48.05
6.	1061	*γ*-Terpinene	0.72
7.	1102	Linalool	2.37
8.	1183	Terpinen-4-ol	3.03
9.	1196	*α*-terpineol	1.85
10.	1289	Bornyl acetate	1.02
11.	1319	Δ-terpinyl acetate	0.88
12.	1359	*α*-terpinyl acetate	13.36
13.	1365	Eugenol	2.24
14.	1407	Methyleugenol	3.04
15.	1587	Spathulenol	0.64
		**Total**	**96.55**
	**Black Pepper Essential Oil**		
1.	940	*α*-pinene	10.56
2.	984	*β*-pinene	13.16
3.	994	Myrcene	3.10
4.	1014	*α*-phellandrene	5.05
5.	1018	*α*-terpinene	24.71
6.	1037	Limonene	19.90
7.	1092	*α*-terpinolene	0.89
8.	1343	Δ-elemene	1.17
9.	1383	Copaene	1.17
10.	1435	*β*-caryophyllene	17.59
11.	1490	Germacrene D	1.06
12.	1504	*α*-selinene	0.82
		**Total**	**99.17**

* Retention indices relative to a series of C8–C20 *n*-alkanes calculated on TRB-5MS column.

**Table 3:** LC-HRMS/MS data of compounds tentatively identified in different spice extracts.

No	Proposed Identity	T_R_ (min)	HRMS	Exp. (*m/z*)	Calcd. (*m/z*)	Δ (ppm)	MF	HRMS/MS (*m/z*)	Extracts
Cinnamon Extracts									
**Ci1**	Quinic acid	1.6	[M-H]^−^	191.0562	191.0561	−0.46	C_7_H_12_O_6_	177.0416, 129.0176, 99.0106	M, MW
**Ci2**	Gluconic acid	1.8	[M-H]^−^	195.0516	195.0510	−2.93	C_6_H_12_O_7_	177.0424, 159.0283, 129.0190, 99.0084	M, MW
**Ci3**	Vanillic acid	2.0	[M-H]^−^	167.0351	167.0350	−0.11	C_8_H_8_O_4_	123.0468	M, MW
**Ci4**	Citric acid	2.2	[M-H]^−^	191.0202	191.0197	−2.47	C_6_H_8_O_7_	129.0202, 111.0090, 87.0085	M, MW
**Ci5**	Ferulic acid	2.3	[M-H]^−^	193.0505	193.0506	0.68	C_10_H_10_O_4_	175.0415, 149.0611	M, MW
**Ci6**	Caffeoylquinic acid	3.0	[M-H]^−^	353.0879	353.0878	−0.27	C_16_H_18_O_9_	191.0622, 179.0435, 173.0515, 135.0428	M, MW
**Ci7**	Hydroxybenzoic acid	3.3	[M-H]^−^	137.0238	137.0244	4.47	C_7_H_6_O_3_	109.0300	M, MW
**Ci8**	(Epi)catechin	3.6	[M-H]^−^	289.0721	289.0718	−1.17	C_15_H_14_O_6_	245.0844, 205.0469, 179.0359	M, MW
**Ci9**	Proanthocyanidin trimer	5.2	[M-H]^−^	863.1817	863.1829	1.37	C_45_H_36_O_18_	711.1326, 573.1002, 531.0845, 451.0996, 411.0654, 289.0700	M, MW
**Ci10**	Coniferol-*O*-hexoside	6.7	[M-H]^−^	341.1241	341.1242	0.27	C_16_H_22_O_8_	179.0572, 161.0588	M, MW
**Ci11**	Trihydroxyoctadecenoic acid	22.1	[M-H]^−^	329.2335	329.2333	−0.46	C_18_H_34_O_5_	229.1440, 211.1313, 171.1011	H, D, M, MW
**Ci12**	Dihydroxyoctadecenoic acid	26.3	[M-H]^−^	313.2396	313.2396	−3.71	C_18_H_34_O_4_	295.2224, 269.1251	H, D, M, MW
**Cumin Extracts**									
**Cu1**	Citric acid	2.0	[M-H]^−^	191.0200	191.0197	−1.43	C_6_H_8_O_7_	173.0083, 129.0188, 111.0089	M, MW
**Cu2**	Caffeoylquinic acid I	2.4	[M-H]^−^	353.0866	353.0878	3.39	C_16_H_18_O_9_	191.0634, 179.0418, 173.0493, 135.0456	M, MW
**Cu3**	Caffeoylquinic acid II	3.0	[M-H]^−^	353.0862	353.0878	4.53	C_16_H_18_O_9_	191.0659, 173.0508, 135.0455	M, MW
**Cu4**	Coniferol-*O*-hexoside	3.3	[M-H]^−^	341.1235	341.1242	2.02	C_16_H_22_O_8_	179.0572, 161.0588	M, MW
**Cu5**	Luteolin-*O*-hexuronide-hexoside	4.6	[M-H]^−^	623.1245	623.1254	1.40	C_27_H_28_O_17_	249.0612, 191.0548, 173.0432	M, MW
**Cu6**	Apigenin-*O*-hexuronide-hexoside	6.9	[M-H]^−^	607.1294	607.1305	1.74	C_27_H_28_O_16_	337.0804, 269.0438	M, MW
**Cu7**	Luteolin-*O*-hexoside	7.9	[M-H]^−^	447.0935	447.0933	−0.48	C_21_H_20_O_11_	285.0383, 169.1920	M, MW
**Cu8**	Apigenin-*O*-hexoside	10.8	[M-H]^−^	431.0973	431.0984	2.48	C_20_H_20_O_10_	269.0417	M, MW
**Cu9**	Luteolin-*O*-acetylhexoside	14.8	[M-H]-	489.1041	489.1038	−0.51	C_23_H_22_O_12_	285.0404, 227.0336	M, MW
**Cu10**	Luteolin	18.4	[M-H]^−^	285.0400	285.0405	1.61	C_15_H_10_O_6_	199.0409, 175.0395, 151.0021, 133.0285	D, M, MW
**Cu11**	Apigenin	22.3	[M-H]^−^	269.0456	269.0455	−0.20	C_15_H_10_O_5_	225.0532, 151.0026, 117.0343	D, M, MW
**Clove Extracts**									
**Cl1**	Gallic acid	1.6	[M-H]^−^	169.0150	169.0142	−4.43	C_7_H_6_O_5_	125.0245	M
**Cl2**	Quinic acid	1.8	[M-H]^−^	191.0554	191.0561	−3.66	C_7_H_12_O_6_	177.0416, 129.0176, 99.0106	M, MW
**Cl3**	Caffeoylquinic acid I	1.5	[M-H]^−^	353.0874	353.0878	−1.13	C_16_H_18_O_9_	191.0640, 179.0444, 135.0493	M
**Cl4**	Citric acid	2.2	[M-H]^−^	191.0192	191.0197	−1.04	C_6_H_8_O_7_	129.0202, 111.0090, 87.0085	M, MW
**Cl5**	Caffeoylquinic acid II	2.4	[M-H]^−^	353.0864	353.0878	3.96	C_16_H_18_O_9_	191.0611, 179.0453, 173.0505, 135.0455	M, MW
**Cl6**	Di-*O*-galloyl-hexahydroxydiphenoyl-hexose	3.3	[M-H]^−^	785.0480	785.0843	0.38	C_34_H_25_O_22_	633.0742, 483.0654, 301.0040, 275.0184	M, MW
**Cl7**	Tri-*O*-galloyl-hexahydroxydiphenoyl-hexose	5.5	[M-H]^−^	937.0951	937.0953	0.16	C_41_H_30_O_26_	767.0704, 741.0940, 635.0813, 599.0655, 571.0686, 465.0626, 419.0584, 300.9977	M, MW
**Cl8**	Ellagic acid	7.9	[M-H]^−^	301.0040	300.9990	−4.67	C_14_H_16_O_8_	284.0000, 275.0113, 257.0113, 245.0097, 229.0169, 217.0167, 201.0212, 185.0264	M, MW
**Cl9**	Quercetin-*O*-hexuronide	9.8	[M-H]^−^	477.0674	477.0675	0.13	C_21_H_18_O_13_	301.0345, 283.0276, 151.0017	M, MW
**Cl10**	Syringic acid-*O*-hexuronide	10.4	[M-H]^−^	373.0778	373.0776	−0.44	C_15_H_18_O_11_	355.0656, 265.0344,193.0091, 167.0318	M, MW
**Cl1**	Isorhamnetin-*O*-hexoside	15.4	[M-H]^−^	477.1034	477.1038	0.94	C_22_H_22_O_12_	315.0490, 299.0159, 271.0198	M, MW
**Cl2**	Isorhamnetin-*O*-hexuronide	17.5	[M-H]^−^	491.0820	491.0831	2.26	C_22_H_20_O_13_	315.0500, 300.0280, 165.0163	M, MW
**Cl13**	Luteolin	18.4	[M-H]^−^	285.0402	285.0405	0.91	C_15_H_10_O_6_	199.0409, 175.0395, 151.0021, 133.0285	M, MW
**Cl14**	Quercetin	18.5	[M-H]^−^	301.0341	301.0354	4.23	C_15_H_10_O_7_	178.9972, 151.007, 107.01201	M, MW
**Cl15**	Isorhamnetin	22.7	[M-H]^−^	315.0508	315.0510	0.72	C_16_H_12_O_7_	300.0258, 151.0026	M, MW
**Cl16**	Chrysoeriol	23.7	[M-H]^−^	299.0557	299.0561	1.37	C_16_H_12_O_6_	284.0303, 275.0499, 255.0289, 227.0378, 151.0027	M, MW
**Cl17**	Cirsiliol	23.8	[M-H]^−^	329.0667	329.0668	−0.07	C_17_H_14_O_7_	314.0416, 271.0296, 243.0249	M, MW
**Cl18**	Trimethylellagic acid	24.3	[M-H]^−^	343.0458	343.0459	−1.04	C_17_H_12_O_8_	328.0193, 297.9724, 285.0032, 269.9768	M, MW
**Cl19**	Cirsimaritin	25.9	[M-H]^−^	313.0715	317.0718	0.83	C_17_H_14_O_6_	298.0489, 193.0110, 151.0035	M, MW
**Laurel Extracts**									
**La1**	Caffeoylquinic acid	1.4	[M-H]^−^	353.0855	353.0878	6.51	C_16_H_18_O_9_	191.0609, 179.0514, 135.0505	M
**La2**	Citric acid	2.2	[M-H]^−^	191.0203	191.0197	3.14	C_6_H_8_O_7_	129.0202, 111.0090, 87.0085	M, MW
**La3**	Gallic acid-*O*-hexoside	4.6	[M-H]^−^	331.0671	331.0671	−0.01	C_13_H_16_O_10_	169.0137, 125.0238	M, MW
**La4**	Proanthocyanidin trimer	4.7	[M-H]^−^	863.1837	863.1829	−0.94	C_45_H_36_O_18_	711.1377, 573.1020, 531.1014, 451.1086, 411.0627, 289.0679	M, MW
**La5**	Quercetin-*O*-deoxyhexoside-hexoside	6.7	[M-H]^−^	609.1468	609.1461	−1.13	C_27_H_30_O_16_	301.0312, 178.9940	M, MW
**La6**	Apigenin-C-hexoside	6.8	[M-H]^−^	431.0989	431.0984	−1.23	C_21_H_20_O_10_	341.0665, 311.0580, 151.0014	M, MW
**La7**	Quercetin-*O*-hexoside	8.1	[M-H]^−^	463.0874	463.0874	1.72	C_21_H_20_O_12_	301.0343, 151.0043	M, MW
**La8**	Isorhamnetin-*O*-deoxyhexoside-hexoside	9.7	[M-H]^−^	623.1617	623.1618	0.90	C_28_H_32_O_16_	315.0449, 299.0208, 150.9978	M, MW
**La9**	Quercetin-*O*-deoxyhexoside	10.7	[M-H]^−^	447.0939	447.0933	−1.37	C_21_H_20_O_11_	301.0371, 271.0303	M, MW
**La10**	Kaempferol-*O*-deoxyhexoside	13.2	[M-H]^−^	431.0987	431.0984	−0.76	C_21_H_20_O_10_	285.0383, 255.0250, 227.0405	M, MW
**La11**	Quercetin	18.1	[M-H]^−^	301.0367	301.0354	−4.38	C_15_H_10_O_7_	178.9972, 151.007, 107.01201	M, MW
**La12**	Kaempferol	22.8	[M-H]^−^	285.0402	285.0405	0.91	C_15_H_10_O_6_	199.0409, 175.0395, 151.0021, 133.0285	M, MW
**La13**	Kaempferol-*O*-coumaroyl-deoxyhexoside	25.9	[M-H]^−^	577.1332	577.1351	3.37	C_30_H_26_O_12_	431.1134, 413.0649, 285.0386, 255.0327, 227.0322	M, MW
**La14**	Kaempferol-di-*O*-coumaroyl-deoxyhexoside I	32.2	[M-H]^−^	723.1711	723.1719	1.28	C_39_H_32_O_14_	577.1336, 437.1228, 413.0865, 285.0391, 255.0300	M, MW
**La15**	Kaempferol-di-*O*-coumaroyl-deoxyhexoside II	33.5	[M-H]^−^	723.1756	723.1719	−5.07	C_39_H_32_O_14_	577.1336, 437.1228, 413.0865, 285.0391, 255.0300	M, MW
**Black Pepper Extracts**									
**Bp1**	Citric acid	2.1	[M-H]^−^	191.0198	191.0197	0.14	C_6_H_8_O_7_	173.0077, 129.0174, 111.0080	M, MW
**Bp2**	Hydroxybenzoic acid	3.3	[M-H]^−^	137.0234	137.0244	7.37	C_7_H_6_O_3_	119.0233, 108.0204	D, M, MW
**Bp3**	Dihydropiperlongumine	10.8	[M+H]^+^	320.1508	320.1492	−4.86	C_17_H_21_NO_5_	302.1417, 274.1427, 217.0479, 189.0547, 170.1177, 152.1067, 112.0789	M, MW
**Bp4**	*N*-Feruloyltyramine	14.9	[M+H]^+^	314.1409	314.1387	−7.08	C_18_H_19_NO_4_	177.0396, 145.0159, 121.0542	H, D, M, MW
**Bp5**	*N*-Coumaroyltyramine	17.3	[M+H]^+^	284.1268	284.1281	4.66	C_17_H_17_NO_3_	177.0516, 145.0262	M, W
**Bp6**	Piperlonguminine	18.5	[M+H]^+^	274.1422	274.1438	5.75	C_16_H_19_NO_3_	203.0688, 189.0523, 147.0425	D, M, MW
**Bp7**	Piperettine I	20.9	[M+H]^+^	312.1567	312.1594	8.74	C_19_H_21_NO_3_	229.0755, 201.0807, 161.0532, 137.0536	H, D, M, MW
**Bp8**	Piperanine	23.3	[M+H]^+^	288.1596	288.1594	−0.63	C_17_H_21_NO_3_	203.0691, 137.0589	H, D, M, MW
**Bp9**	Piperyline	26.1	[M+H]^+^	272.1301	272.1281	−6.93	C_16_H_17_NO_3_	201.0569, 173.0609, 135.0414	H, D, M, MW
**Bp10**	Dehyropiperettine	28.8	[M+H]^+^	310.1437	310.1380	0.23	C_19_H_19_NO_3_	227.06523, 135.0453	H, D, M, MW
**Bp11**	Piperine	30.2	[M+H]^+^	286.1440	286.1438	−0.81	C_17_H_19_NO_3_	201.0542, 173.0504, 135.0414	H, D, M, MW
**Bp12**	Piperdardine	31.8	[M+Na]^+^	336.1582	336.1570	−3.78	C_19_H_23_NO_3_	174.0839, 160.0734	H, D, M, MW
**Bp13**	Piperettine II	32.4	[M+H]^+^	312.1602	312.1594	−2.51	C_19_H_21_NO_3_	227.0694, 199.0748, 169.0638	H, D, M
**Bp14**	Piperettine III	34.8	[M+H]^+^	312.1575	312.1594	6.17	C_19_H_21_NO_3_	227.0684, 199.0730, 112.0747	H, D, M, MW
**Bp15**	Piperettine IV	35.4	[M+H]^+^	312.1612	312.1594	−5.72	C_19_H_21_NO_3_	227.0609, 199.0735, 169.0640, 112.0747	H, D, M
**Bp16**	Pellitorine	36.8	[M+H]^+^	224.2006	224.2009	1.31	C_14_H_25_NO	168.1360, 123.1144	H, D, M, MW
**Bp17**	Pipercallosine	37.4	[M+H]^+^	330.2085	330.2064	−6.47	C_20_H_27_NO_3_	208.1724, 135.0456	H, D, M, MW
**Bp18**	Neopellitorine B	38.1	[M+H]^+^	236.1987	236.2009	9.32	C_15_H_25_NO	151.1111, 112.0771	H, D, M
**Bp19**	Dehydropipernonaline	38.5	[M+H]^+^	340.1923	340.1907	−4.66	C_21_H_25_NO_3_	201.1127, 135.0647	H, D, M, MW
**Bp20**	Pipernonaline	39.5	[M+H]^+^	342.2084	342.2064	−5.95	C_21_H_27_NO_3_	229.1241, 199.1119, 161.0614, 135.0448	H, D, M, MW
**Bp21**	Piperolein B	41.1	[M+H]^+^	344.2199	344.2220	6.18	C_21_H_29_NO_3_	222.1830, 135.0429	H, D, M, MW
**Bp22**	Retrofractamide B	42.5	[M+H]^+^	356.2196	356.2220	6.81	C_22_H_29_NO_3_	255.1341, 234.1848, 135.1148	H, D, M, MW
**Bp23**	*N*-Isobutyl-dodecadienamide	43.2	[M+H]^+^	252.2334	252.2322	−4.81	C_16_H_29_NO	196.1679, 152.1049	H, D, M
**Bp24**	Piperchabamide B	45.1	[M+H]^+^	370.2389	370.2377	−3.33	C_23_H_31_NO_3_	285.1521, 135.0449, 112.0758	H, D, M, MW
**Bp25**	Brachyamide A	45.9	[M+H]^+^	382.2384	382.2377	−1.91	C_24_H_31_NO_3_	283.1686, 135.0437	H, D, M, MW
**Bp26**	Pipgulzarine	46.7	[M+H]^+^	372.2515	372.2533	4.90	C_23_H_33_NO_3_	250.2171, 135.0432	H, D, M, MW
**Bp27**	Guineensine	47.4	[M+H]^+^	384.2513	384.2533	4.28	C_24_H_33_NO_3_	311.1632, 283.1698, 187.0762, 161.0603	H, D, M, MW
**Bp28**	Piperflaviflorine	48.6	[M+H]^+^	386.2717	386.2690	−7.08	C_24_H_35_NO_3_	313.1786, 285.1840, 264.2304, 161.0584	H, D, M
**Bp29**	Piperchabamide C	49.1	[M+H]^+^	396.2518	396.2533	3.85	C_25_H_33_NO_3_	283.1668, 135.0426	H, D, M
**Bp30**	Pipwaqarine	49.5	[M+H]^+^	398.2697	398.269	−1.84	C_25_H_35_NO_3_	311.1683, 283.1689, 161.0601, 135.0440	H, D, M

Abbreviations: Bp—black pepper; Ci—cinnamon; Cl—clove; Cu—cumin; D—dichloromethane extract; H—hexane extract; HRMS—high-resolution mass spectrometry; La—laurel; M—methanol extract; MF—molecular formula; MW—50% aqueous methanol extract; T_R_—retention time; Δ—mass error.

**Table 4:** TPC and TFC of different spice extracts.

Sample	TPC	TFC
	(mg GAE/g)	(mg RE/g)
**Cinnamon Extracts**		
**CiH**	18.46 ± 0.27 ^c^	3.88 ± 0.02 ^b^
**CiD**	14.00 ± 0.15 ^d^	3.27 ± 0.14 ^c^
**CiMW**	92.90 ± 0.46 ^a^	1.76 ± 0.11 ^d^
**CiM**	63.68 ± 1.48 ^b^	9.83 ± 0.09 ^a^
**Cumin Extracts**		
**CuH**	7.86 ± 0.11 ^d^	0.48 ± 0.05 ^cd^
**CuD**	9.75 ± 0.07 ^d^	0.72 ± 0.05 ^c^
**CuMW**	30.77 ± 0.38 ^a^	66.25 ± 0.33 ^a^
**CuM**	19.69 ± 0.54 ^b^	50.68 ± 0.41 ^b^
**Clove Extracts**		
**ClH**	100.86 ± 0.59 ^b^	1.43 ± 0.06 ^d^
**ClD**	90.67 ± 1.24 ^d^	5.89 ± 0.12 ^c^
**ClMW**	103.76 ± 0.23 ^a^	41.71 ± 0.13 ^a^
**ClM**	94.46 ± 0.53 ^c^	31.00 ± 0.25 ^b^
**Laurel Extracts**		
**LaH**	22.49 ± 0.31 ^d^	0.50 ± 0.07 ^c^
**LaD**	24.29 ± 0.18 ^c^	0.55 ± 0.02 ^c^
**LaMW**	75.53 ± 0.10 ^a^	19.50 ± 0.25 ^b^
**LaM**	48.95 ± 0.32 ^b^	27.33 ± 0.40 ^a^
**Black Pepper Extracts**		
**BpH**	31.50 ± 0.14 ^d^	6.78 ± 0.59 ^c^
**BpD**	33.51 ± 0.10 ^c^	8.64 ± 0.35 ^b^
**BpMW**	37.01 ± 0.15 ^a^	2.31 ± 0.21 ^d^
**BpM**	35.42 ± 0.13 ^b^	9.67 ± 0.28 ^a^

Data are presented as mean ± standard deviation (SD) of three determinations; different superscript letters within columns indicate significant differences in the tested extracts for the same species (*p* < 0.05). Abbreviations: BpD—black pepper dichloromethane extract;; BpH—black pepper hexane extract; BpM—black pepper methanol extract; BpMW—black pepper 50% aqueous methanol extract; CiD—cinnamon dichloromethane extract; CiH—cinnamon hexane extract; CiM—cinnamon methanol extract; CiMW—cinnamon 50% aqueous methanol extract; ClD—clove dichloromethane extract; ClH—clove hexane extract; ClM—clove methanol extract; ClMW—clove 50% aqueous methanol extract; CuD—cumin dichloromethane extract; CuH—cumin hexane extract; CuM—cumin methanol extract; CuMW—cumin 50% aqueous methanol extract; GAE—gallic acid equivalents; LaD—laurel dichloromethane extract; LaH—laurel hexane extract; LaM—laurel methanol extract; LaMW—laurel 50% aqueous methanol extract; RE—rutin equivalents; TFC—total flavonoid content; TPC—total phenolic content.

**Table 5:** Antioxidant activity of different spice extracts and essential oils.

Sample	DPPH	ABTS	CUPRAC	FRAP	MCA	PBD
	(mg TE/g)				(mg EDTAE/g)	(mmol TE/g)
**Cinnamon Extracts**						
**CiH**	6.96 ± 0.41 ^d^	21.03 ± 0.55 ^d^	21.17 ± 0.25 ^e^	10.60 ± 1.05 ^e^	20.46 ± 0.37 ^a^	1.66 ± 0.09 ^c^
**CiD**	9.39 ± 0.57 ^d^	20.01 ± 0.55 ^d^	47.98 ± 0.42 ^d^	26.54 ± 4.83 ^d^	4.86 ± 0.13 ^c^	1.22 ± 0.04 ^d^
**CiMW**	473.74 ± 1.45 ^c^	588.97 ± 6.34 ^c^	623.44 ± 5.09 ^b^	380.73 ± 4.96 ^b^	19.72 ± 0.60 ^ab^	2.14 ± 0.08 ^b^
**CiM**	178.42 ± 0.81 ^a^	235.58 ± 2.04 ^b^	257.93 ± 8.49 ^c^	168.96 ± 7.49 ^c^	16.31 ± 0.43 ^b^	1.67 ± 0.04 ^c^
**CiEO**	210.00 ± 2.39 ^b^	839.94 ± 1.89 ^a^	762.89 ± 13.47 ^a^	834.77 ± 1.46 ^a^	18.20 ± 4.51 ^ab^	18.26 ± 0.29 ^a^
**Cumin Extracts**						
**CuH**	0.44 ± 0.03 ^d^	5.07 ± 0.36 ^d^	22.77 ± 0.27 ^d^	14.81 ± 2.17 ^d^	4.82 ± 0.41 ^c^	0.60 ± 0.06 ^c^
**CuD**	1.53 ± 0.05 ^c^	5.29 ± 0.28 ^d^	17.57 ± 0.86 ^e^	12.54 ± 2.81 ^d^	n.a.	0.61 ± 0.01 ^c^
**CuMW**	46.39 ± 0.28 ^a^	77.14 ± 0.09 ^a^	118.66 ± 3.06 ^a^	82.60 ± 0.46 ^b^	26.96 ± 0.27 ^a^	0.99 ± 0.01 ^b^
**CuM**	44.62 ± 0.08 ^b^	36.94 ± 0.23 ^b^	67.12 ± 2.19 ^c^	48.01 ± 2.09 ^c^	14.22 ± 0.85 ^b^	1.04 ± 0.03 ^b^
**CuEO**	n.a.	13.69 ± 2.05 ^c^	105.21 ± 1.71 ^b^	87.71 ± 0.97 ^a^	n.a.	40.33 ± 3.62 ^a^
**Clove Extracts**						
**ClH**	394.48 ± 1.37 ^d^	727.19 ± 1.00 ^c^	866.12 ± 47.11 ^c^	1209.92 ± 71.14 ^b^	3.64 ± 0.30^c^	6.02 ± 0.08 ^b^
**ClD**	355.23 ± 1.44 ^e^	669.32 ± 4.71 ^d^	753.39 ± 2.65 ^d^	973.49 ± 5.96 ^c^	4.09 ± 0.79 ^c^	5.21 ± 0.06 ^b^
**ClMW**	488.45 ± 0.38 ^b^	770.12 ± 1.40 ^b^	978.08 ± 21.03 ^b^	1027.41 ± 12.79 ^c^	22.78 ± 1.31 ^a^	4.92 ± 0.01 ^b^
**ClM**	437.36 ± 3.59 ^c^	722.91 ± 1.91 ^c^	778.93 ± 8.46 ^d^	876.30 ± 13.24 ^d^	14.71 ± 0.33 ^b^	4.38 ± 0.11 ^b^
**ClEO**	525.78 ± 0.30 ^a^	936.44 ± 0.17 ^a^	2848.28 ± 13.89 ^a^	1927.98 ± 18.80 ^a^	11.88 ± 1.87 ^b^	68.19 ± 3.25 ^a^
**Laurel Extracts**						
**LaH**	2.08 ± 0.16 ^e^	11.40 ± 0.64 ^e^	28.58 ± 4.44 ^e^	14.11 ± 2.89 ^e^	14.50 ± 0.63 ^c^	2.02 ± 0.09 ^b^
**LaD**	6.27 ± 0.45 ^d^	21.52 ± 1.35 ^d^	52.90 ± 2.63 ^d^	32.34 ± 2.84 ^d^	21.22 ± 0.84 ^b^	2.07 ± 0.10 ^b^
**LaMW**	194.14 ± 0.48 ^a^	280.91 ± 0.77 ^b^	303.47 ± 7.16 ^b^	196.49 ± 2.92 ^b^	14.83 ± 0.50 ^c^	2.03 ± 0.02 ^b^
**LaM**	113.12 ± 1.56 ^b^	143.84 ± 12.76 ^c^	143.40 ± 1.03 ^c^	93.23 ± 7.95 ^c^	16.32 ± 0.35 ^bc^	1.99 ± 0.09 ^b^
**LaEO**	91.57 ± 1.33 ^c^	507.33 ± 6.15 ^a^	416.67 ± 21.48 ^a^	435.97 ± 4.23 ^a^	33.78 ± 5.03 ^a^	49.10 ± 2.53 ^a^
**Black Pepper Extracts**						
**BpH**	18.77 ± 0.24 ^d^	41.41 ± 1.67 ^c^	52.39 ± 0.80 ^c^	68.19 ± 0.31 ^c^	20.62 ± 2.13 ^b^	1.35 ± 0.03 ^b^
**BpD**	19.56 ± 0.59 ^c^	34.26 ± 0.58 ^d^	48.96 ± 4.87 ^c^	53.69 ± 6.64 ^d^	10.94 ± 0.26 ^d^	0.98 ± 0.02 ^c^
**BpMW**	45.41 ± 0.03 ^a^	76.58 ± 0.13 ^a^	106.38 ± 0.45 ^b^	84.06 ± 1.61 ^b^	30.51 ± 0.11 ^a^	1.09 ± 0.00 ^c^
**BpM**	32.41 ± 0.07 ^b^	49.88 ± 0.88 ^b^	51.22 ± 1.64 ^c^	68.27 ± 3.19 ^c^	n.a.	0.95 ± 0.02 ^c^
**BpEO**	n.a.	33.75 ± 3.55 ^d^	174.06 ± 2.57 ^a^	169.06 ± 2.23 ^a^	15.72 ± 0.59 ^c^	59.80 ± 6.50 ^a^

Data are presented as mean ± standard deviation (SD) of three determinations; different superscript letters within columns indicate significant differences in the tested extracts for the same species (*p* < 0.05). Abbreviations: ABTS—2,2′-azino-bis(3-ethylbenzothiazoline) 6-sulfonic acid; BpD—black pepper dichloromethane extract; BpEO—black pepper essential oil; BpH—black pepper hexane extract; BpM—black pepper methanol extract; BpMW—black pepper 50% aqueous methanol extract; CiD—cinnamon dichloromethane extract; CiEO—cinnamon essential oil; CiH—cinnamon hexane extract; CiM—cinnamon methanol extract; CiMW, cinnamon 50% aqueous methanol extract; ClD, clove dichloromethane extract; ClEO, clove essential oil; ClH—clove hexane extract; ClM—clove methanol extract; ClMW—clove 50% aqueous methanol extract; CuD—cumin dichloromethane extract; CuEO—cumin essential oil; CuH—cumin hexane extract; CuM—cumin methanol extract; CuMW—cumin 50% aqueous methanol extract; CUPRAC—cupric ion reducing antioxidant capacity; DPPH—1,1-diphenyl-2-picrylhydrazyl; EDTAE—EDTA equivalents; FRAP—ferric ion reducing antioxidant power; LaD—laurel dichloromethane extract; LaEO—laurel essential oil; LaH—laurel hexane extract; LaM—laurel methanol extract; LaMW—laurel 50% aqueous methanol extract; MCA—metal chelating activity; n.a.—not active; PBD—phosphomolybdenum assay; TE—Trolox equivalents.

**Table 6:** Anti-enzymatic activity of different spice extracts and essential oils.

Sample	AChE	BChE	Tyrosinase	Amylase	Glucosidase
	(mg GALAE/g)		(mg KAE/g)	(mmol ACAE/g)	
**Cinnamon Extracts**					
**CiH**	1.70 ± 0.02 ^b^	1.95 ± 0.00 ^b^	18.24 ± 4.89 ^b^	0.53 ± 0.01 ^a^	0.88 ± 0.00 ^a^
**CiD**	1.14 ± 0.03 ^c^	1.87 ± 0.07 ^b^	23.88 ± 0.54 ^b^	0.41 ± 0.02 ^c^	0.88 ± 0.00 ^a^
**CiMW**	1.75 ± 0.05 ^b^	0.45 ± 0.05 ^c^	66.45 ± 0.32 ^a^	0.41 ± 0.02 ^c^	n.a.
**CiM**	2.01 ± 0.20 ^a^	3.18 ± 0.05 ^a^	61.56 ± 3.53 ^a^	0.45 ± 0.02 ^b^	0.80 ± 0.00 ^b^
**CiEO**	1.77 ± 0.17 ^b^	n.a.	24.41 ± 4.10 ^b^	n.a.	n.a.
**Cumin Extracts**					
**CuH**	2.95 ± 0.01 ^a^	2.57 ± 0.02 ^a^	26.31 ± 2.63 ^d^	0.31 ± 0.03 ^a^	0.82 ± 0.02 ^a^
**CuD**	2.34 ± 0.09 ^b^	2.11 ± 0.05 ^b^	28.30 ± 1.60 ^c^	0.33 ± 0.02 ^a^	0.84 ± 0.00 ^a^
**CuMW**	1.04 ± 0.04 ^d^	n.a.	63.12 ± 0.51 ^a^	0.30 ± 0.00 ^a^	0.45 ± 0.01 ^c^
**CuM**	1.53 ± 0.07 ^c^	n.a.	58.05 ± 2.65 ^a^	0.31 ± 0.02 ^a^	0.77 ± 0.01 ^b^
**CuEO**	0.73 ± 0.02 ^e^	n.a.	41.31 ± 4.83 ^b^	n.a.	n.a.
**Clove Extracts**					
**ClH**	2.48 ± 0.35 ^ab^	2.45 ± 0.22 ^b^	20.79 ± 0.73 ^c^	0.32 ± 0.03 ^b^	0.83 ± 0.00 ^c^
**ClD**	1.96 ± 0.30 ^bc^	1.97 ± 0.17 ^c^	22.51 ± 0.63 ^c^	0.32 ± 0.01 ^b^	0.84 ± 0.01 ^c^
**ClMW**	1.64 ± 0.11 ^b^	n.a.	59.23 ± 0.14 ^a^	0.19 ± 0.01 ^c^	n.a.
**ClM**	1.96 ± 0.11 ^bc^	0.58 ± 0.02 ^d^	40.33 ± 4.09 ^b^	0.30 ± 0.02 ^b^	0.89 ± 0.00 ^b^
**ClEO**	2.77 ± 0.18 ^a^	2.94 ± 0.09 ^a^	38.16 ± 5.16 ^b^	0.68 ± 0.01 ^a^	1.00 ± 0.02 ^a^
**Laurel Extracts**					
**LaH**	2.47 ± 0.12 ^a^	3.41 ± 0.63 ^a^	n.a.	0.55 ± 0.00 ^b^	0.80 ± 0.02 ^b^
**LaD**	1.59 ± 0.01 ^bc^	1.68 ± 0.33 ^b^	n.a.	0.57 ± 0.01 ^b^	0.77 ± 0.01 ^c^
**LaMW**	1.04 ± 0.03 ^d^	1.44 ± 0.33 ^b^	43.38 ± 1.53 ^a^	0.32 ± 0.00 ^c^	0.89 ± 0.01 ^a^
**LaM**	1.82 ± 0.08 ^b^	1.66 ± 0.05 ^b^	27.68 ± 1.56 ^b^	0.45 ± 0.01 ^bc^	0.83 ± 0.01 ^b^
**LaEO**	1.35 ± 0.15 ^e^	0.41 ± 0.10 ^c^	22.48 ± 0.59 ^c^	1.29 ± 0.13 ^a^	n.a.
**Black Pepper Extracts**					
**BpH**	1.48 ± 0.03 ^c^	n.a.	76.86 ± 1.29 ^a^	1.00 ± 0.09 ^a^	0.85 ± 0.00 ^a^
**BpD**	2.55 ± 0.03 ^a^	1.24 ± 0.32 ^b^	69.56 ± 2.44 ^b^	0.99 ± 0.02 ^a^	0.76 ± 0.02 ^b^
**BpMW**	0.76 ± 0.04 ^d^	2.46 ± 0.22 ^a^	49.31 ± 1.12 ^c^	0.58 ± 0.01 ^c^	0.17 ± 0.00 ^c^
**BpM**	1.78 ± 0.05 ^b^	1.26 ± 0.02 ^b^	43.94 ± 1.25 ^d^	0.84 ± 0.06 ^b^	0.75 ± 0.01 ^b^
**BpEO**	2.43 ± 0.10 ^a^	2.16 ± 0.03 ^a^	26.43 ± 0.36 ^e^	n.a.	n.a.

Data are presented as mean ± standard deviation (SD) of three determinations; different superscript letters within columns indicate significant differences in the tested extracts for the same species (*p* < 0.05). Abbreviations: ACAE—acarbose equivalents; AChE—acetylcholinesterase; BChE—butyrylcholinesterase; BpD—black pepper dichloromethane extract; BpEO—black pepper essential oil; BpH—black pepper hexane extract; BpM—black pepper methanol extract; BpMW—black pepper 50% aqueous methanol extract; CiD—cinnamon dichloromethane extract; CiEO—cinnamon essential oil; CiH—cinnamon hexane extract; CiM—cinnamon methanol extract; CiMW—cinnamon 50% aqueous methanol extract; ClD—clove dichloromethane extract; ClEO—clove essential oil; ClH—clove hexane extract; ClM—clove methanol extract; ClMW—clove 50% aqueous methanol extract; CuD—cumin dichloromethane extract; CuEO—cumin essential oil; CuH—cumin hexane extract; CuM—cumin methanol extract; CuMW—cumin 50% aqueous methanol extract; GALAE—galanthamine equivalents; KAE—kojic acid equivalants; LaD—laurel dichloromethane extract; LaEO—laurel essential oil; LaH—laurel hexane extract; LaM—laurel methanol extract; LaMW—laurel 50% aqueous methanol extract; n.a.—not active.

## Data Availability

Not applicable.
